# 4-(4-Nitro­phen­yl)morpholine

**DOI:** 10.1107/S1600536812012172

**Published:** 2012-03-31

**Authors:** Li-Jiao Wang, Wei-Wei Li, Sheng-Yong Yang, Li Yang

**Affiliations:** a State Key Laboratory of Biotherapy and Cancer Center, West China Hospital, West China Medical School, Sichuan University, Chengdu 610041, People’s Republic of China; b Department of Applied Chemistry, College of Chemical Engineering, Sichuan University, Chengdu 610041, People’s Republic of China

## Abstract

Aromatic π–π stacking inter­actions stabilize the crystal structure of the title compound, C_10_H_12_N_2_O_3_, the perpendic­ular distance between parallel planes being 3.7721 (8) Å. The morpholine ring adopts a chair comformation.

## Related literature

For the biological activity and synthesis of 4-(4-nitro­phen­yl)morpholine derivatives, see: Wang *et al.* (2010 ▶). For a related structure, see: Yang *et al.* (2011 ▶).
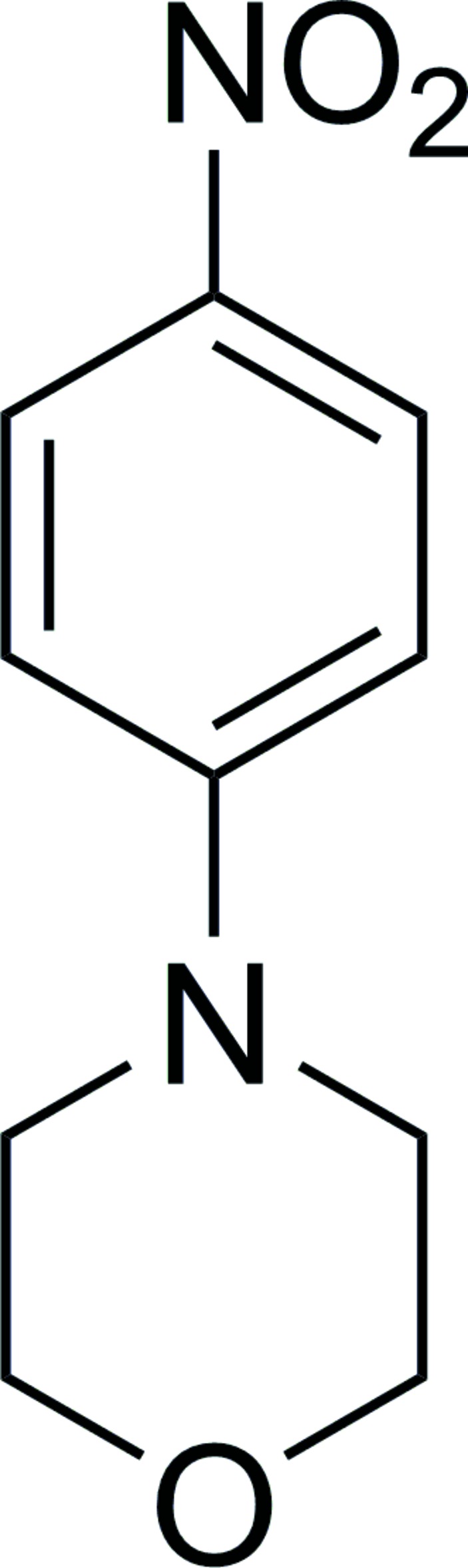


## Experimental

### 

#### Crystal data

C_10_H_12_N_2_O_3_
*M* *_r_* = 208.22
Orthorhombic, 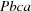

*a* = 14.5445 (6) Å
*b* = 8.3832 (3) Å
*c* = 16.2341 (6) Å
*V* = 1979.42 (13) Å^3^
*Z* = 8
Mo *K*α radiation
μ = 0.11 mm^−1^
*T* = 293 K
0.35 × 0.33 × 0.30 mm

#### Data collection

Oxford Diffraction Xcalibur Eos diffractometer
Absorption correction: multi-scan (*CrysAlis PRO*; Oxford Diffraction, 2006 ▶) *T*_min_ = 0.992, *T*_max_ = 1.000
4949 measured reflections
2023 independent reflections
1377 reflections with *I* > 2σ(*I*)
*R*_int_ = 0.018

#### Refinement

*R*[*F* ^2^ > 2σ(*F* ^2^)] = 0.048
*wR*(*F* ^2^) = 0.121
*S* = 1.03
2023 reflections
184 parameters
All H-atom parameters refined
Δρ_max_ = 0.12 e Å^−3^
Δρ_min_ = −0.15 e Å^−3^

### 

Data collection: *CrysAlis PRO* (Oxford Diffraction, 2006 ▶); cell refinement: *CrysAlis PRO*; data reduction: *CrysAlis PRO*; program(s) used to solve structure: *SHELXS97* (Sheldrick, 2008 ▶); program(s) used to refine structure: *SHELXL97* (Sheldrick, 2008 ▶); molecular graphics: *OLEX2* (Dolomanov *et al.*, 2009 ▶); software used to prepare material for publication: *OLEX2*.

## Supplementary Material

Crystal structure: contains datablock(s) global, I. DOI: 10.1107/S1600536812012172/kj2195sup1.cif

Structure factors: contains datablock(s) I. DOI: 10.1107/S1600536812012172/kj2195Isup2.hkl

Supplementary material file. DOI: 10.1107/S1600536812012172/kj2195Isup3.cml

Additional supplementary materials:  crystallographic information; 3D view; checkCIF report

## Figures and Tables

**Fig. 1.**
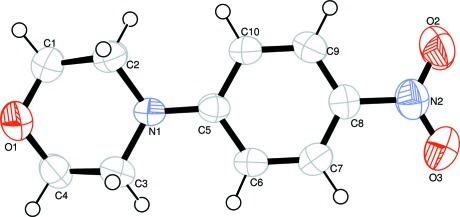
The molecular structure of the title compound, with displacement ellipsoids drawn at the 30% probability level.

**Fig. 2.**
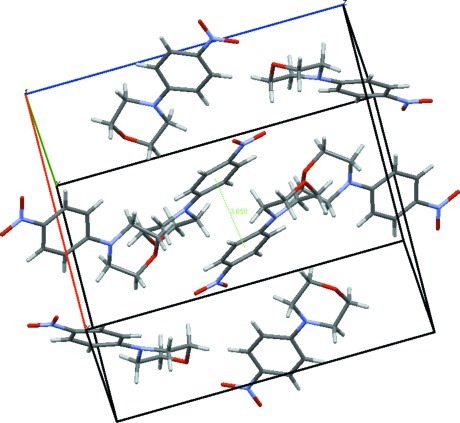
A packing diagram of the title compound. The dotted line indicates the Cg—Cg(-x,-y,1 - z) distance.

## supplementary crystallographic information

### Comment

4-(4-nitrophenyl)morpholine derivatives are of great importance due to their anticancer activity (Wang *et al.*, 2010;). The title compound is one of the key intermediates in our synthetic investigations of antitumor drugs. We synthesized the title compound and report its crystal structure in this paper.

In the title compound, C_10_H_12_N_2_O_3_, (Fig. 1) the bond lengths and angles are within normal ranges (Yang *et al.*, 2011). Aromatic π–π stacking interactions help to stabilize the crystal structure (Fig. 2). The perpendicular distance between the parallel ring planes is 3.7721 (8) Å, the distance between the centres of gravity *Cg*—*Cg*(-*x*,-*y*,1 - *z*) is 3.8499 (11) Å.

### Experimental

The title compound was prepared by a method similar to that of Shudong Wang *et al.* (2010), which Crystals suitable for X-ray analysis were obtained by slow evaporation from a solution of dichloromethane.

### Refinement

All H atoms were positioned in the difference map and refined freely.

### Crystal data

**Table:**

C_10_H_12_N_2_O_3_	*D*_x_ = 1.397 Mg m^−^^3^
*M**_r_* = 208.22	Mo *K*α radiation, λ = 0.71073 Å
Orthorhombic, *P**b**c**a*	Cell parameters from 1704 reflections
*a* = 14.5445 (6) Å	θ = 2.9–29.2°
*b* = 8.3832 (3) Å	µ = 0.11 mm^−^^1^
*c* = 16.2341 (6) Å	*T* = 293 K
*V* = 1979.42 (13) Å^3^	Block, yellow
*Z* = 8	0.35 × 0.33 × 0.30 mm
*F*(000) = 880	

### Data collection

**Table:**

Oxford Diffraction Xcalibur Eos diffractometer	2023 independent reflections
Radiation source: Enhance (Mo) X-ray Source	1377 reflections with *I* > 2σ(*I*)
Graphite monochromator	*R*_int_ = 0.018
Detector resolution: 16.0874 pixels mm^-1^	θ_max_ = 26.4°, θ_min_ = 2.9°
ω scans	*h* = −9→18
Absorption correction: multi-scan (*CrysAlis PRO*; Oxford Diffraction, 2006)	*k* = −6→10
*T*_min_ = 0.992, *T*_max_ = 1.000	*l* = −20→12
4949 measured reflections	

### Refinement

**Table:**

Refinement on *F*^2^	Primary atom site location: structure-invariant direct methods
Least-squares matrix: full	Secondary atom site location: difference Fourier map
*R*[*F*^2^ > 2σ(*F*^2^)] = 0.048	Hydrogen site location: difference Fourier map
*wR*(*F*^2^) = 0.121	All H-atom parameters refined
*S* = 1.03	*w* = 1/[σ^2^(*F*_o_^2^) + (0.050*P*)^2^ + 0.3012*P*] where *P* = (*F*_o_^2^ + 2*F*_c_^2^)/3
2023 reflections	(Δ/σ)_max_ < 0.001
184 parameters	Δρ_max_ = 0.12 e Å^−^^3^
0 restraints	Δρ_min_ = −0.15 e Å^−^^3^

### Special details

**Table:**

Geometry. All e.s.d.'s (except the e.s.d. in the dihedral angle between two l.s. planes) are estimated using the full covariance matrix. The cell e.s.d.'s are taken into account individually in the estimation of e.s.d.'s in distances, angles and torsion angles; correlations between e.s.d.'s in cell parameters are only used when they are defined by crystal symmetry. An approximate (isotropic) treatment of cell e.s.d.'s is used for estimating e.s.d.'s involving l.s. planes.
Refinement. Refinement of *F*^2^ against ALL reflections. The weighted *R*-factor *wR* and goodness of fit *S* are based on *F*^2^, conventional *R*-factors *R* are based on *F*, with *F* set to zero for negative *F*^2^. The threshold expression of *F*^2^ > σ(*F*^2^) is used only for calculating *R*-factors(gt) *etc*. and is not relevant to the choice of reflections for refinement. *R*-factors based on *F*^2^ are statistically about twice as large as those based on *F*, and *R*- factors based on ALL data will be even larger.

### Fractional atomic coordinates and isotropic or equivalent isotropic displacement parameters (Å^2^)

**Table:**

	*x*	*y*	*z*	*U*_iso_*/*U*_eq_	
O1	0.11977 (11)	0.40333 (15)	0.24876 (9)	0.0774 (5)	
O2	0.15361 (12)	−0.3154 (2)	0.66760 (10)	0.0931 (6)	
O3	0.09389 (13)	−0.47156 (17)	0.57725 (10)	0.0907 (6)	
N1	0.12607 (10)	0.15429 (16)	0.36653 (8)	0.0488 (4)	
N2	0.12312 (11)	−0.3406 (2)	0.59853 (11)	0.0642 (5)	
C1	0.17590 (18)	0.4172 (3)	0.31932 (13)	0.0674 (6)	
H1A	0.2408 (16)	0.378 (2)	0.3051 (12)	0.083 (7)*	
H1B	0.1775 (14)	0.531 (2)	0.3339 (12)	0.072 (6)*	
C2	0.14099 (17)	0.3205 (2)	0.39042 (13)	0.0587 (5)	
H2A	0.1869 (14)	0.327 (2)	0.4354 (12)	0.067 (6)*	
H2B	0.0823 (14)	0.367 (2)	0.4102 (12)	0.068 (6)*	
C3	0.07821 (15)	0.1361 (3)	0.28780 (11)	0.0567 (5)	
H3A	0.0113 (15)	0.159 (2)	0.2958 (12)	0.081 (7)*	
H3B	0.0813 (13)	0.028 (2)	0.2697 (11)	0.064 (6)*	
C4	0.11879 (17)	0.2413 (2)	0.22354 (13)	0.0647 (5)	
H4A	0.0814 (13)	0.237 (2)	0.1743 (13)	0.072 (6)*	
H4B	0.1848 (14)	0.205 (2)	0.2122 (12)	0.077 (6)*	
C5	0.12154 (11)	0.03660 (19)	0.42504 (10)	0.0440 (4)	
C6	0.08684 (14)	−0.1153 (2)	0.40613 (12)	0.0589 (5)	
H6	0.0618 (13)	−0.137 (2)	0.3546 (12)	0.069 (6)*	
C7	0.08671 (14)	−0.2364 (2)	0.46268 (12)	0.0598 (5)	
H7	0.0634 (14)	−0.340 (2)	0.4490 (12)	0.078 (6)*	
C8	0.12173 (12)	−0.2108 (2)	0.54007 (11)	0.0501 (4)	
C9	0.15440 (14)	−0.0625 (2)	0.56225 (12)	0.0563 (5)	
H9	0.1773 (13)	−0.045 (2)	0.6160 (13)	0.065 (6)*	
C10	0.15375 (13)	0.0592 (2)	0.50585 (11)	0.0536 (5)	
H10	0.1772 (13)	0.161 (2)	0.5228 (11)	0.064 (5)*	

### Atomic displacement parameters (Å^2^)

**Table:**

	*U*^11^	*U*^22^	*U*^33^	*U*^12^	*U*^13^	*U*^23^
O1	0.1061 (12)	0.0596 (8)	0.0664 (9)	0.0081 (8)	−0.0178 (9)	0.0111 (7)
O2	0.1083 (13)	0.0960 (12)	0.0748 (11)	−0.0164 (10)	−0.0257 (10)	0.0297 (9)
O3	0.1163 (13)	0.0583 (9)	0.0974 (12)	−0.0137 (9)	−0.0058 (10)	0.0163 (8)
N1	0.0586 (9)	0.0459 (7)	0.0418 (8)	−0.0054 (7)	−0.0048 (7)	−0.0049 (6)
N2	0.0576 (10)	0.0686 (11)	0.0665 (11)	0.0035 (9)	0.0008 (9)	0.0131 (9)
C1	0.0868 (16)	0.0538 (12)	0.0615 (13)	−0.0091 (12)	−0.0020 (12)	0.0044 (10)
C2	0.0720 (13)	0.0498 (10)	0.0544 (11)	−0.0060 (10)	0.0003 (11)	−0.0067 (9)
C3	0.0624 (13)	0.0599 (12)	0.0477 (11)	−0.0024 (10)	−0.0086 (9)	−0.0048 (9)
C4	0.0829 (15)	0.0636 (12)	0.0478 (11)	0.0061 (12)	−0.0116 (11)	0.0029 (9)
C5	0.0428 (9)	0.0475 (8)	0.0418 (9)	−0.0013 (8)	0.0019 (7)	−0.0054 (7)
C6	0.0722 (13)	0.0569 (11)	0.0476 (11)	−0.0143 (10)	−0.0093 (10)	−0.0055 (9)
C7	0.0682 (12)	0.0497 (10)	0.0617 (12)	−0.0106 (10)	−0.0015 (10)	−0.0030 (9)
C8	0.0467 (9)	0.0521 (9)	0.0514 (10)	0.0024 (8)	0.0028 (8)	0.0037 (8)
C9	0.0626 (12)	0.0622 (11)	0.0443 (10)	−0.0015 (9)	−0.0044 (9)	−0.0042 (8)
C10	0.0656 (11)	0.0495 (9)	0.0458 (10)	−0.0081 (9)	−0.0035 (9)	−0.0069 (8)

### Geometric parameters (Å, º)

**Table:**

O1—C1	1.411 (2)	C3—H3B	0.958 (19)
O1—C4	1.418 (2)	C3—C4	1.488 (3)
O2—N2	1.224 (2)	C4—H4A	0.97 (2)
O3—N2	1.227 (2)	C4—H4B	1.02 (2)
N1—C2	1.463 (2)	C5—C6	1.404 (2)
N1—C3	1.463 (2)	C5—C10	1.406 (2)
N1—C5	1.371 (2)	C6—H6	0.93 (2)
N2—C8	1.444 (2)	C6—C7	1.369 (3)
C1—H1A	1.03 (2)	C7—H7	0.96 (2)
C1—H1B	0.98 (2)	C7—C8	1.373 (3)
C1—C2	1.499 (3)	C8—C9	1.378 (2)
C2—H2A	0.99 (2)	C9—H9	0.95 (2)
C2—H2B	0.99 (2)	C9—C10	1.371 (3)
C3—H3A	1.00 (2)	C10—H10	0.957 (18)
			
C1—O1—C4	108.61 (15)	O1—C4—C3	111.68 (18)
C2—N1—C3	113.67 (15)	O1—C4—H4A	106.5 (11)
C5—N1—C2	120.60 (14)	O1—C4—H4B	109.0 (11)
C5—N1—C3	120.47 (14)	C3—C4—H4A	109.4 (11)
O2—N2—O3	122.50 (17)	C3—C4—H4B	108.9 (11)
O2—N2—C8	118.51 (17)	H4A—C4—H4B	111.5 (16)
O3—N2—C8	118.98 (17)	N1—C5—C6	121.23 (15)
O1—C1—H1A	108.8 (12)	N1—C5—C10	122.24 (15)
O1—C1—H1B	106.8 (11)	C6—C5—C10	116.50 (16)
O1—C1—C2	112.60 (18)	C5—C6—H6	121.2 (12)
H1A—C1—H1B	110.1 (17)	C7—C6—C5	121.78 (18)
C2—C1—H1A	108.2 (12)	C7—C6—H6	117.0 (12)
C2—C1—H1B	110.3 (12)	C6—C7—H7	121.1 (12)
N1—C2—C1	111.18 (17)	C6—C7—C8	119.81 (18)
N1—C2—H2A	110.4 (11)	C8—C7—H7	119.1 (12)
N1—C2—H2B	109.4 (11)	C7—C8—N2	119.25 (17)
C1—C2—H2A	108.0 (11)	C7—C8—C9	120.55 (17)
C1—C2—H2B	109.2 (11)	C9—C8—N2	120.20 (17)
H2A—C2—H2B	108.6 (16)	C8—C9—H9	120.3 (11)
N1—C3—H3A	109.2 (12)	C10—C9—C8	119.62 (18)
N1—C3—H3B	110.2 (11)	C10—C9—H9	120.1 (11)
N1—C3—C4	111.20 (16)	C5—C10—H10	120.4 (11)
H3A—C3—H3B	105.7 (16)	C9—C10—C5	121.68 (17)
C4—C3—H3A	111.2 (12)	C9—C10—H10	117.9 (11)
C4—C3—H3B	109.2 (11)		
